# Alcohol Use Disorder in the Age of Technology: A Review of Wearable Biosensors in Alcohol Use Disorder Treatment

**DOI:** 10.3389/fpsyt.2021.642813

**Published:** 2021-03-22

**Authors:** Rachel E. Davis-Martin, Sheila M. Alessi, Edwin D. Boudreaux

**Affiliations:** ^1^Department of Emergency Medicine, University of Massachusetts Medical School, Worcester, MA, United States; ^2^Department of Medicine, Calhoun Cardiology Center, University of Connecticut School of Medicine, Farmington, CT, United States

**Keywords:** wearable biosensors, alcohol use disorder, treatment, transdermal alcohol content, contingency management

## Abstract

Biosensors enable observation and understanding of latent physiological occurrences otherwise unknown or invasively detected. Wearable biosensors monitoring physiological constructs across a wide variety of mental and physical health conditions have become an important trend in innovative research methodologies. Within substance use research, explorations of biosensor technology commonly focus on identifying physiological indicators of intoxication to increase understanding of addiction etiology and to inform treatment recommendations. In this review, we examine the state of research in this area as it pertains to treatment of alcohol use disorders specifically highlighting the gaps in our current knowledge with recommendations for future research. Annually, alcohol use disorders affect approximately 15 million individuals. A primary focus of existing wearable technology-based research among people with alcohol use disorders is identifying alcohol intoxication. A large benefit of wearable biosensors for this purpose is they provide continuous readings in a passive manner compared with the gold standard measure of blood alcohol content (BAC) traditionally measured intermittently by breathalyzer or blood draw. There are two primary means of measuring intoxication with biosensors: gait and sweat. Gait changes have been measured via smart sensors placed on the wrist, in the shoe, and mobile device sensors in smart phones. Sweat measured by transdermal biosensors detects the presence of alcohol in the blood stream correlating to BAC. Transdermal biosensors have been designed in tattoos/skin patches, shirts, and most commonly, devices worn on the ankle or wrist. Transdermal devices were initially developed to help monitor court-ordered sobriety among offenders with alcohol use disorder. These devices now prove most useful in continuously tracking consumption throughout clinical trials for behavioral treatment modalities. More recent research has started exploring the uses for physical activity trackers and physiological arousal sensors to guide behavioral interventions for relapse prevention. While research has begun to demonstrate wearable devices' utility in reducing alcohol consumption among individuals aiming to cutdown on their drinking, monitoring sustained abstinence in studies exploring contingency management for alcohol use disorders, and facilitating engagement in activity-based treatment interventions, their full potential to further aid in understanding of, and treatment for, alcohol use disorders has yet to be explored.

## Introduction

In the United States, each year, alcohol use disorders (AUD) affect roughly 15 million individuals and cost nearly $250 billion in treatment and economic losses (1, 2). An estimated 7% of people who need treatment for AUD ever receive it resulting in staggering mortality and morbidity (2). Treatment options include outpatient therapy, intensive outpatient programs, and inpatient admission with detoxification, with each approach increasing in costs, patient time demands, and disrupting quality of life. Advances in technology have the potential to significantly improve these barriers to treatment (3). Research using novel technology to address public health problems has increased exponentially in the twenty first century. Broadly known as digital health, this area of research explores the use of mobile and computer software applications and wearable biosensor devices to better understand and treat health conditions (4–9). Within research on AUD, the use of biosensors specifically has become an important trend in innovative research methodologies (3). Biosensor devices enable observation and understanding of latent physiological occurrences otherwise unknown or invasively detected. Wearable biosensors are placed externally on the body and typically measure movement, sweat or skin conductance, heart rate, or temperature (9). In AUD biosensor research literature, devices tested fall into two categories: (1) those designed specifically to detect alcohol use (e.g., identifying intoxication) and (2) those designed for primarily other purposes (e.g., physical activity) but are being explored in specific use cases related to alcohol. The former has a significantly larger body of literature, with most research over the past 20 years centering on creating and refining biosensors to detect intoxication in laboratory and field-testing environments (10–13).

Recent reviews on wearable biosensors for alcohol use have provided in-depth looks into the mechanics of how specific biosensors work (14), the status of biosensor development ranging from prototype to commercial availability (15, 16), the benefits and pitfalls of different devices (14, 16), and implications for how numerous sensors can be used to improve research on pharmacotherapy and interactions with alcohol use (17). In this review, we will summarize the use of wearable biosensors in clinical research of AUD treatments. We will (1) provide a brief introduction to the various types of alcohol-specific biosensor devices currently available that hold potential for uses in AUD treatment research, (2) examine in-depth how wearables devices are being used in clinical research to improve existing or provide novel approaches to AUD treatment, and (3) highlight the gaps in current knowledge and consider implications for future research. The overall goal of this review is to examine how researchers are utilizing existing wearable technologies in treatments for AUD.

## Measuring Intoxication

There are two primary means of measuring intoxication with biosensors: gait and sweat. Gait is measured by locomotion on three axes—forward/backward, side-to-side, up and down, and ground reaction forces—the impact of the force of the foot as it hits the ground. Using machine learning algorithms, patterns of movement are mapped onto known blood alcohol content (BAC) levels measured by blood draws or breathalyzer to better understand how people move at various levels of intoxication. A recent development in locomotion research with alcohol has even mapped patterns of movement with machine learning algorithms to determine when someone is holding and lifting the drink and how long they are taking sips (17). Gait-tracking biosensors can be worn on the ankle and wrist, inserted in shoes, or detected by mobile phones typically in the pocket. In addition to standard gait measurements, with the use of mobile phones, patterns of engagement with mobile applications (e.g., rate of typing, number of errors made while typing) on the phones can also be used to infer intoxication (18). Research using gait to identify alcohol use first emerged in the literature in 2012 (19), with the bulk of research published within the past 5 years (20–26). The diverse design and placement of gait-tracking biosensors may facilitate a burgeoning body of exploration among AUD treatment samples; however, to date, research using these biosensors occurs exclusively in non-treatment samples and contexts. The most common research sample is among college students, a group known for their risky drinking behaviors, and the most common purpose is to prevent drinking and driving (27). The nascent status of using gait measuring devices and complex algorithms to understand alcohol use likely contributes to why these types of sensors have yet to be used in AUD treatment research.

Sweat, including interstitial fluid that exists between cells containing sweat glands, is measured by transdermal devices that electronically analyze biomarkers in vapors on the surface of the skin and secretions inside the dermis. As alcohol emanates from the bloodstream and diffuses through the skin, transdermal devices detect the amount of alcohol in sweat and interstitial fluid that then algorithmically correlates to BAC measured by blood draw or breathalyzer (28–30). Additional algorithms have been tested to further align transdermal alcohol levels with standard drink units (30). Since the chemical makeup of sweat can differ for each individual, measuring transdermal alcohol content requires a baseline reading absent of any consumed alcohol. Transdermal alcohol readings have approximately a 30-min lag between consumption and detection compared with breath-based readings (28–30). Biosensors measuring changes in skin temperature and heart rate variability can supplement sweat and gait data to improve detection of alcohol consumption (17). Coupled with GPS-based location data, these physiological data points can be used to determine proximity to places where someone might socially consume alcohol or purchase alcohol. While many of the sweat-based devices have been validated using samples of people in AUD treatment, the focus of those studies was not treatment related, but rather ensuring that the devices accurately captured higher levels of BAC seen in this population compared with the community. In treatment studies using transdermal devices, the purposes focus on (1) comparing findings to traditional alcohol use measurement (i.e., patient self-report or use of breathalyzer, blood draws, or urine analysis), (2) using the alcohol use data to inform intervention efforts, or (3) assessing acceptability and feasibility of using the device while in AUD treatment. Table 1 provides an overview of devices measuring gait and sweat to detect alcohol intoxication.

**Table 1:** Biosensor devices measuring alcohol intoxication.

**Device**	**Location**	**Intoxication measurement**	**Testing status/Availability**
SCRAM®	Ankle	Transdermal alcohol content	Commercially available
Giner WrisTAS^TM^	Wrist	Transdermal alcohol content	• Completed laboratory testing • Completed several field tests • Not currently commercially available
BACtrack Skyn^TM^	Wrist	Transdermal alcohol content	• Completed laboratory testing • Currently in field testing • Commercial availability anticipated 2021
Proof^TM^	Wrist	Transdermal alcohol content	Discontinued after laboratory testing
Quantac Tally	Wrist	Transdermal alcohol content	Discontinued after laboratory testing
Iontophoretic-biosensing system	Tattoo on arm	Transdermal alcohol content	Currently in laboratory testing (31)
AlcoWear	Wrist + phone	Gait smartphone AlcoGait application paired with any smart watch to measure accelerometer and gyroscope	Currently in laboratory testing
Sensor-equipped smart shoes	Shoes	Gait using pressure sensors inserted in insole of shoe	Currently in laboratory testing
AlcoGait	Phone	Gait using smartphone application to gather accelerometer and gyroscope	Currently in laboratory testing
DrinkTRAC	Phone	Gait using smartphone application to gather accelerometer, gyroscope, and magnetometer	Currently in laboratory testing

## Alcohol-Specific Biosensors

The Secure Continuous Remote Alcohol Monitor (SCRAM®) is the most widely used wearable device in clinical research trials and has been adopted internationally by justice systems for court-monitored sobriety. The SCRAM® is an ankle worn monitor that electrochemically measures transdermal alcohol content. The device takes measurements roughly once every half hour and more frequently when a heavy drinking episode is detected or tampering is suspected. The measurements result in three alcohol-related outcomes: (1) total transdermal alcohol content reported as grams of alcohol per deciliter of sweat (g/dl), (2) absorption rate or how quickly alcohol appears in the sweat reported as g/dl per hour, and (3) elimination rate or how quickly alcohol leaves the sweat also reported as g/dL per hour. The measurements are stored on the device and uploaded via wired or wireless connection onto a secure web-based server that provides reports on transdermal alcohol and other measurements. The device can be worn continuously and contains features that deter removal and that detect potential tampering, which triggers an alert and investigation to confirm/disprove.

Technology like SCRAM® has made it possible to advance AUD research and treatment in ways not previously possible. For example, contingency management (CM) is an efficacious substance use disorder treatment that has been difficult to apply to AUD because of the need to objectively verify abstinence. Contingency management, based on principles of learning and behavioral economics, is a program of systematically reinforcing (incentivizing) objective evidence of pro-health target behaviors to improve outcomes, such as reinforcing abstinence to improve substance use disorder treatment outcomes. There is robust evidence supporting improvements in abstinence and harm reduction with CM (32–34). Few studies have assessed CM for AUD because the most common objective measure of alcohol consumption is the alcohol breath test, which has a small window of detection (hours) and thus usually requires either several in-person tests per day to detect all use, or if used clinically, occurs too infrequently to detect alcohol use between treatment sessions. Now, transdermal alcohol sensing technology allows for remote objective monitoring of alcohol use near continuously, and CM research incorporating this technology is occurring.

Barnett et al. (35) conducted the first pilot using wearable biosensors with a CM intervention to change alcohol consumption behaviors. This study utilized a sample of 13 individuals in the community who reported heavy drinking habits (≥8 drinks per week for women, ≥15 for men) with an interest in reducing alcohol consumption. Participants wore the SCRAM® monitor and self-reported alcohol use for 3 weeks. The first week was an observation period where no CM intervention was provided, and drinking patterns were just reported. In weeks 2 and 3 of the study, monetary reinforcement was provided for each day when no alcohol use was detected by SCRAM®. Alcohol use detection was defined by one transdermal alcohol reading >0.02 g/dl, and either an absorption rate <0.05 g/dl per hour for a single drinking episode or an elimination rate <0.025 g/dl per hour (when peak <0.15 g/dl) and > 0.035 g/dl per hour (when peak >0.15 g/dl) for a single drinking episode. For the initial day with no alcohol use detected, participants received $5, and each consecutive day per week with no alcohol detection, monetary reinforcement increased by $2, consistent with CM best practices and research (36). At the end of the 3-week study period, there were significant increases in the number of days abstinent in week 2 and 3 compared with the 1-week observation period (*p* < 0.001) and significant reductions in the average transdermal alcohol content across all days indicating a reduction in the number of drinks consumed each sitting (*p* < 0.01). Barnett et al. (37) built upon these findings by conducting a second study among a similar sample (*N* = 30) using the same CM schedule and SCRAM® criteria for earning monetary reinforcement. In addition to the drinks per week inclusion criteria used in the aforementioned study, this study also required individuals to have two or more heavy drinking episodes per week (>3 for women, >4 for men in one sitting) to be eligible to participate. Furthermore, the CM intervention period was increased from 2 to 3 weeks, and they added a 1-month follow-up period to track progress once CM was removed. During the follow-up period, participants did not wear the SCRAM® bracelet and self-reported alcohol use during that timeframe. Participants were randomized to either a CM group or a non-contingent reinforcement group (controls) in which monetary compensation was yoked to reinforcement earned by a matched CM participant and not contingent on drinking status. Compared with the controls, participants in the CM group had a higher percentage of device-derived abstinent days (*p* = 0.05) and longer periods of device-derived consecutive days without drinking detected (*p* = 0.03) during the 3 intervention weeks. At the end of the 1 month follow-up period, there were no differences between groups on the number of consecutive days without drinking. These findings suggest that monetary incentives linked to transdermal alcohol readings can reduce risky alcohol consumption while incentives are available.

Dougherty et al. (38–40) also used SCRAM® in several CM studies aimed at reducing the amount of alcohol consumed weekly by community-based heavy drinkers defined as >3 drinks for women or >4 drinks for men in one sitting at least three times in the last 28 days. Unlike the aforementioned Barnett et al. (35) study, these studies used a harm reduction approach, meaning that rather than requiring abstinence, participants received monetary reinforcement for days with alcohol consumption not exceeding 0.03 g/dl levels on three consecutive SCRAM® measurements. This threshold was chosen as transdermal levels > 0.03 g/dl typically correspond to light to moderate drinking (one to two beers) (28). In the first study, 26 at-risk drinkers (defined above) were randomized to one of two groups that varied in the timing of CM for scientific purposes. When provided, monetary reinforcement for each group was delivered once a week and was earned when participants did not exceed 0.03 g/dl in any single day throughout the past week. In Group A, participants received no monetary reinforcement for 4 weeks followed by 4 weeks of $25 monetary reinforcement per week of no drinking detected. In Group B, the sequence of the reinforcement was switched, with participants receiving $25 monetary reinforcement in the first 4 weeks followed by 4 weeks with no monetary reinforcement. After 8 weeks, both groups then received weekly monetary reinforcements of $50 for an additional 4 weeks. Results showed significant reductions in heavy drinking days during the 8-week CM periods compared with the 4 weeks with no monetary reinforcement (*p* < 0.001). Furthermore, drinking reductions did not differ between the $25 per week phase and $50 per week phase, suggesting that the lower cost program may be a viable option for reducing heavy drinking (38). Dougherty's et al. (39, 40) second study expanded upon the abovementioned findings by increasing the length of CM period from 2 to 3 months and adding a follow-up period to track progress once CM was removed. In this study, 82 participants wore SCRAM® for a 4-week observation period followed by a 12-week period with weekly delivered $50 monetary reinforcement for not exceeding 0.03 g/dl in any single day throughout the past week. At the end of the 12-week CM period, the SCRAM® monitor was removed, and participants were followed up once monthly for 3 months to self-report alcohol use in the past 28 days. SCRAM® data demonstrated that participants were seven times more likely to not exceed the 0.03 g/dl drinking limit during the CM period compared with the observation period (*p* = 0.01). Additionally, using self-report data, the participants had significantly fewer heavy and moderate (adjusted for sex) drinking days during both the 12-week CM and 3-month follow-up period compared with the observation period (*p* < 0.001), with participants reporting similar drinking behaviors during the CM period and the 3 months following discontinuation of CM (39, 40). Unlike the conclusions from Barnett et al. (37), this study suggests that reductions in heavy alcohol consumption with CM for alcohol-negative transdermal alcohol readings may continue after removal of incentives. The mixed findings may be attributable to reinforcing abstinence vs. drinking reductions. Another possible contributing factor is the duration or other parameters of CM prior to discontinuation of incentives. Future research is needed to better understand the mixed results.

Two studies have reported on SCRAM® among AUD treatment patients (41, 42). In both studies, all participants received standard community-based AUD treatment services including group therapy, 12-Step, and relapse prevention approaches. Alessi et al. (42) reported on the feasibility and acceptability of monitoring from the initial 100 participants in one of two randomized control trials of 12-weeks of SCRAM® monitoring of alcohol consumption and CM for either AUD treatment attendance or CM for each day of SCRAM®-based no drinking among community-based outpatient AUD treatment patients. They found that relatively few patients declined to participate in the study because of the SCRAM® monitoring aspect (<10%), and the majority (84%) provided SCRAM® data every day of the 12-week treatment period. Most participants reported neutral comfort with the bracelet, denied the bracelet interfered with their mood, sleep, work, and normal activities, and believed the bracelet helped them reduce their drinking. These findings indicate that SCRAM® may be feasibly incorporated into outpatient treatment of AUD. Additionally, Alessi et al. (41) used SCRAM® to track alcohol use for 3 months in 63 patients in community-based AUD treatment. SCRAM® was used to objectively and near-continuously characterize individuals' drinking habits while in outpatient treatment for the first time. Participants also completed self-reports of drinking about twice weekly (for research purposes only, and not shared with clinic staff), and device data were compared with self-reports to assess the level of agreement. The SCRAM® data indicated a higher percentage of patients who drank any alcohol and drank heavily compared with the percentage who drank and drank heavily per self-reports. These results suggest that wearable biosensors may provide clinically useful information to supplement self-reports, which can be biased by response demands and other contextual factors (42–44). Throughout the study, clinicians were not provided access to SCRAM® data; thus, they were not able to use this information to inform treatment decisions.

## Physical Activity Biosensors

In addition to wearable devices explicitly designed for alcohol use, researchers have begun using biosensors originally developed for other purposes to improve treatments of alcohol use disorder. Abrantes et al. (45) tested the feasibility of a novel physical activity intervention using FitBit Charge watches aimed to prevent relapse in woman with depression going through alcohol treatment. Twenty women were enrolled from a partial hospitalization program and were given FitBit Charges and provided counseling on the benefits of physical activity on managing cravings and difficult emotions. Participants were given a list of free/low-cost activities that they could engage in throughout the 12-week intervention and were each given a daily step goal that increased by 500 steps each week of the intervention. Once discharged from the 10-day partial hospitalization program, they were offered 6, 30-min phone calls from an activity counselor who addressed barriers to engaging in physical activity and tailored step goals to meet their individual needs. Five women did not complete the intervention, and analyses were conducted using both intention-to-treat and among completers only to address missing data. Similar results were seen in both analytic approaches. Among completers only, 44% were abstinent of alcohol throughout the entire intervention, and on average, women were abstinent on 95% of the days during the 12 weeks. Participants reported a significant increase in use of physical activity to improve mood and cope with urges to drink compared with at the start of the study (*p* < 0.05). These results show promise for using physical activity monitored by biosensors to increase coping strategies for cravings to drink alcohol and maintain abstinence in those critical months following discharge from a partial hospitalization program.

Similarly, Linke et al. (46) conducted a feasibility study among 11 veterans in outpatient substance use treatment aimed to reduce alcohol and illicit drug use through a physical activity program monitored by FitBit HR (47). Participants completed a 12-week intervention that provided weekly psychoeducation groups focused on integrating physical activity for a healthy lifestyle while reducing substance use and a YMCA membership with access to group training sessions and a Fit4Me personal training program. Each participant was given the exercise-related goals of 150+ min per week of aerobic exercise, 2–3 weekly strength training activities, and at least 2 weekly flexibility sessions. Once a week, participants met with a study clinician to review the FitBit HR data and address any barriers to engaging in physical activity. At the end of the 12-week intervention, participants reported a significant change in the number of days using alcohol each month and number of drinks per day when drinking (*p* < 0.001). Participants reported in qualitative interviews that the FitBit HR helped them be accountable to their physical activity plan, and that seeing daily and weekly progress on their physical activity goals helped them stay motivated to remain abstinent of alcohol and other drugs.

## Emotion-Focused Biosensors

Leonard et al. (47) used the Empatica E4 wristband to monitor electrodermal activity (e.g., stress or emotional arousal) in a feasibility study of a mobile application-based mindfulness intervention for college women referred from the university counseling center for problem drinking defined as scoring three or higher on the Alcohol Use Disorders Identification Test—Consumption (AUDIT-C). Participants (*N* = 10) in the study wore the E4 wristband for 1 week before receiving an in-person counseling session that assessed several factors related to drinking behaviors (e.g., readiness to change, consequences of drinking, short-term goals) and their stress and physical activity in the past week, and setup an alert to activate when the E4 wristband reached an individually determined stress threshold. For 3 weeks following this session, participants were instructed to use the Mind the Moment mobile application when they received the stress-related alert. The application guides users through brief cognitive behavioral and mindfulness strategies (e.g., deep breathing, meditation, and positive self-talk). Participants were also encouraged to use the application between E4 stress-related alerts. In qualitative interviews, 70% of the sample stated that using the application and wearing the wristband helped them reduce risky drinking behaviors and adhere to their short-term drinking goals set in the counseling session. The purpose of the study was on the feasibility of using a mobile application-based intervention paired with a wearable biosensor, and quantitative data on changes in number of drinks per week/month were not reported.

Additionally, Rouw (48) developed and tested a treatment protocol integrating Sense-IT biosensor data and associated mobile application into inpatient alcohol treatment to enable awareness of physiological processes elicited by arousal, emotions, and cravings. Sense-IT is an ambulatory biofeedback mobile application that utilizes heart rate data from a smartwatch to provide haptic and visual feedback on wearer's heart rate. The biosensor plus associated mobile application was originally created for patients with Borderline Personality Disorder to improve emotion awareness and regulation (49). As this specific-use case of Sense-IT was novel, Rouw explored both providers' (*N* = 3) and patients' (*N* = 2) input on the potential value of integrating the technology into existing treatment modalities. For COVID-related logistical reasons, Rouw tested this technology among addiction treatment inpatients as opposed to ambulatory care patients as initially planned. For 3 weeks, patient participants completed structured sessions using guided imagery and Sense-IT biofeedback with the goal of increasing user's awareness of physiological arousal that occurs when experiencing cravings to drink. During the biofeedback sessions, participants were given cognitive behavioral therapeutic interventions to cope with the cravings and see the impact of those interventions on their physiological arousal. Patient participants reported they found added value in using Sense-IT to better understand their cravings and added that it facilitated conversations with the interventionist about strategies for coping with cravings. They noted that the technology may be more useful in an outpatient setting where access to treatment providers is less constant, and the environment is less controlled. Provider participants also reported that the Sense-IT could be a useful tool in improving patients' awareness of their cravings and cueing them to take therapeutic action rather than use substances.

## Discussion

In treatment studies utilizing wearable biosensors, participants generally report good feasibility and acceptability of the devices, suggesting that integration into treatment may be acceptable among patients. Among the limited number of studies using wearable technology in AUD treatment contexts, the majority used these devices to measure alcohol use or abstinence throughout study periods. With treatments like contingency management, using wearable devices eliminates the need for participants to undergo invasive blood draws or use a breathalyzer during their sessions, which may be more desirable. Furthermore, recent studies are beginning to expand biosensor uses to include incorporating physiological data, like heart rate and number of steps, into the treatment modality. While these approaches represent advancements with potential to improve AUD treatment, applications to date have been narrow in scope. The time is ripe for expansion of novel uses for wearable biosensors in this domain of clinical research.

There are several important areas related to using wearable technology to treat individuals with AUD that are yet to be explored. One is incorporating feedback from transdermal alcohol content biosensors into other treatment modalities besides contingency management. Biosensor data could replace or supplement self-reported alcohol use in behavioral and cognitive behavioral treatments for AUD and inform more accurate and tailored treatment recommendations. For example, based on harm reduction principles, with real-time feedback on alcohol levels, clinicians and patients in an outpatient setting could collaboratively set a daily transdermal alcohol content goal for 1 week at a time with expectations for reducing those levels to either abstinent or healthy levels over time rather than abrupt cessation or abstinence. Additionally, clinicians could review the biosensor data with the patient and help them understand specific drinking patterns (e.g., quantity, rate of consumption, and time of consumption) in relation to specific environmental and social factors and tailor treatment recommendations and coping strategies based on this data. Incorporating feedback on alcohol use could similarly be used with pharmacotherapies that treat AUD. Baseline recordings of transdermal alcohol content could potentially help guide dosing decisions for initiation of medication-assisted therapy. Furthermore, continuous monitoring of numerous physiological constructs using an additional biosensor like FitBit throughout pharmacotherapy could highlight interaction effects of alcohol and other substances on the effectiveness of the medication-assisted therapy (17).

Incorporating wearable alcohol biosensors into Just-In-Time Adaptive Interventions (JITAIs) to reduce drinking is another area for future investigation. These interventions aim to monitor dynamic states (e.g., of risk of substance use) and provide support of the right kind, at the right time, and only when needed. Examples in this growing literature include alcohol research that combined smartphone sensor data and ambulatory self-reports of alcohol use to build machine learning models to predict future high-risk drinking (50). In other work, natural language processing of online recovery forums and machine learning techniques were used to build models to automatically detect potential recovery problems, for potential for intervention (51). A meta-analysis of JITAI studies (e.g., on diet, mental health, addiction, and blood glucose control) found moderate-to-large effect sizes with JITAIs compared with control conditions and non-JITAI interventions (52). Thus, research suggests that incorporating alcohol biosensors into the development of effective JITAIs has great potential to advance delivering clinically meaningful support to individuals where and when needed.

Potential effectiveness of JITAIs could be further enhanced by using biosensors to better understand and detect craving: a known phenomenon in individuals with moderate to severe alcohol use disorder that lacks consensus on definition, factors influencing it, and assessment (53, 54). Craving is marked by several physiological (e.g., heart rate, blood pressure, sweat, saliva) and emotional (e.g., anxiety and dysregulation) changes that biosensor data and complex machine learning algorithms may be able to measure and quantify in a clinically significant way. Intervening in real-time when an individual is experiencing cravings has important implications for preventing relapse, particularly in the early stages of treatment (54, 55). While the use of biosensors to detect cravings may have the greatest synergy with JITAIs, they could easily be incorporated in a number of treatment modalities to improve treatment outcomes, including medication-assisted therapies targeting craving.

An additional area of significant potential is in the treatment of alcohol withdrawal syndrome. Though many of the commercially available devices are not specifically designed to measure withdrawal symptoms, they are capable of measuring many physiological symptoms that occur in alcohol withdrawal (e.g., variable heart rate, proximal sweats, and changes in temperature). Combining a transdermal alcohol content device with an additional biosensor that measures these physiological changes could create an automated detection for alcohol withdrawal symptoms that drastically improves upon the existing measurement and treatment of alcohol withdrawal, including protracted withdrawal where symptoms can persist for many months subsequent to the immediate period following abrupt cessation (56). Furthermore, devices that measure withdrawal symptoms could facilitate an outpatient treatment protocol for detoxification, reducing the treatment burden of lengthy inpatient stays. Following detoxification, the biosensors could then be used in some of the aforementioned potential treatment modalities for monitoring and informing outpatient treatment.

In conclusion, wearable biosensors have demonstrated their utility in improving delivery of cost-effective, evidence-based treatments for AUD and are currently being explored in novel ways to further improve AUD treatment options and access. There are numerous innovative uses of wearable biosensors yet to be explored in AUD treatment, and future research should tap into this potential.

## Author Contributions

RD-M conducted the literature review, composed majority of the paper, created table, and compiled references. SA contributed unique content in the review and discussion sections and expert revisions on content provided by RD-M. EB provided guidance on subject matter, revisions on flow of paper, and iterative edits to content written by RD-M. All authors contributed to the article and approved the submitted version.

## Conflict of Interest

The authors declare that the research was conducted in the absence of any commercial or financial relationships that could be construed as a potential conflict of interest.
